# Do heart and respiratory rate variability improve prediction of extubation outcomes in critically ill patients?

**DOI:** 10.1186/cc13822

**Published:** 2014-04-08

**Authors:** Andrew JE Seely, Andrea Bravi, Christophe Herry, Geoffrey Green, André Longtin, Tim Ramsay, Dean Fergusson, Lauralyn McIntyre, Dalibor Kubelik, Donna E Maziak, Niall Ferguson, Samuel M Brown, Sangeeta Mehta, Claudio Martin, Gordon Rubenfeld, Frank J Jacono, Gari Clifford, Anna Fazekas, John Marshall

**Affiliations:** 1 Ottawa Hospital Research Institute, 725 Parkdale Avenue, Ottawa, ON K1Y 4E9, Canada; 2 University of Ottawa, 75 Laurier Avenue East, Ottawa, ON K1N 6N5, Canada; 3 University Hospital Network, University of Toronto, 190 Elizabeth Street, Toronto, ON M5G 2C4, Canada; 4 Intermountain Medical Center (IMC), Shock Trauma ICU, 5121 Cottonwood Street, Murray, UT 84157, USA; 5 Mt Sinai, University of Toronto, 600 University Avenue, Toronto, ON M5G 1X5, Canada; 6 London Health Sciences Center, 339 Windermere Road, London, ON N6G 2V4, Canada; 7 Sunnybrook Hospital, University of Toronto, 2075 Bayview Avenue, Toronto, ON M4N 3M5, Canada; 8 University Hospital Case Medical Center, Case Western Reserve University, 11100 Euclid Avenue, Cleveland, OH 44106, USA; 9 University of Oxford, Kellogg College, Banbury Road, Oxford OX2 6PN United Kingdom; 10 St. Michaels Hospital, University of Toronto, 30 Bond Street, Toronto, ON M5B 1W8, Canada; 11 Divisions of Thoracic Surgery & Critical Care Medicine, 501 Smyth Road, Ottawa, ON K1H 8L6, Canada

## Abstract

**Introduction:**

Prolonged ventilation and failed extubation are associated with increased harm and cost. The added value of heart and respiratory rate variability (HRV and RRV) during spontaneous breathing trials (SBTs) to predict extubation failure remains unknown.

**Methods:**

We enrolled 721 patients in a multicenter (12 sites), prospective, observational study, evaluating clinical estimates of risk of extubation failure, physiologic measures recorded during SBTs, HRV and RRV recorded before and during the last SBT prior to extubation, and extubation outcomes. We excluded 287 patients because of protocol or technical violations, or poor data quality. Measures of variability (97 HRV, 82 RRV) were calculated from electrocardiogram and capnography waveforms followed by automated cleaning and variability analysis using Continuous Individualized Multiorgan Variability Analysis (CIMVA™) software. Repeated randomized subsampling with training, validation, and testing were used to derive and compare predictive models.

**Results:**

Of 434 patients with high-quality data, 51 (12%) failed extubation. Two HRV and eight RRV measures showed statistically significant association with extubation failure (*P* <0.0041, 5% false discovery rate). An ensemble average of five univariate logistic regression models using RRV during SBT, yielding a probability of extubation failure (called WAVE score), demonstrated optimal predictive capacity. With repeated random subsampling and testing, the model showed mean receiver operating characteristic area under the curve (ROC AUC) of 0.69, higher than heart rate (0.51), rapid shallow breathing index (RBSI; 0.61) and respiratory rate (0.63). After deriving a WAVE model based on all data, training-set performance demonstrated that the model increased its predictive power when applied to patients conventionally considered high risk: a WAVE score >0.5 in patients with RSBI >105 and perceived high risk of failure yielded a fold increase in risk of extubation failure of 3.0 (95% confidence interval (CI) 1.2 to 5.2) and 3.5 (95% CI 1.9 to 5.4), respectively.

**Conclusions:**

Altered HRV and RRV (during the SBT prior to extubation) are significantly associated with extubation failure. A predictive model using RRV during the last SBT provided optimal accuracy of prediction in all patients, with improved accuracy when combined with clinical impression or RSBI. This model requires a validation cohort to evaluate accuracy and generalizability.

**Trial registration:**

ClinicalTrials.gov NCT01237886. Registered 13 October 2010.

## Introduction

The clinical decision to extubate an intensive care unit (ICU) patient is critical to both the quality and efficiency of care. Early extubation is desirable to decrease the risks of prolonged intubation, including progressive respiratory muscle weakness [1], risk of ventilator-associated pneumonia [2], and increased health-care expenditures [3]. Conversely, clinicians aim to limit or avoid failed extubation (usually defined as reintubation within 48 hours of extubation), as it is associated with increased mortality, length of stay, and cost, as well as greater need for long-term rehabilitative care [4,5]. Failed extubation can lead to worse outcomes because of complications that occur at the time of reintubation, especially if performed emergently, including an adverse impact of prolonged intubation, and deterioration prior to reintubation [6]. The mortality risk associated with failed extubation is variable and dependent on the reason for reintubation, with airway obstruction, aspiration, or secretions carrying a lower risk than pneumonia or heart failure [7]. Further compounded by projected increasing costs for care of the critically ill [8], there is a need for improved strategies to reducing the duration of mechanical ventilation while simultaneously avoiding failed extubation [9].

Spontaneous breathing trials (SBTs) - short-duration trials of reduced ventilatory support to simulate the increased work of breathing after extubation - are widely used to evaluate readiness for extubation [10]. A variety of parameters including respiratory rate (RR), tidal volume (TV), rapid shallow breathing index (RSBI = RR/TV or ‘Tobin Index’ [11]), airway pressure during the first 100 ms of inspiration (P0.1), partial pressure of arterial oxygen to fraction of inspired oxygen ratio (P/F), maximal inspiratory or expiratory pressure (MIP or MEP), and cough strength have been evaluated as indicators of extubation readiness [11-13]. In the largest multicenter study of this question, factors that independently increased risk of extubation failure included an elevated RSBI during spontaneous breathing trial (SBT), positive fluid balance and history of pneumonia [13]. Current recommendations for extubation include a 30 to 120 minute SBT during which multiple physiological parameters are used to assess whether the SBT is a pass, fail or equivocal [14]. However, multiple international studies demonstrate that 10 to 15% of ICU patients fail extubation and require reintubation within 48 to 72 hours, with rates between 25 and 30% in high-risk patients [5,11,15,16].

Complex systems analysis has been increasingly used to characterize biological phenomena. The manifestation of complex systems behavior is evident in the high degree and complexity of variability in the time series of inter-beat intervals (that is. interval between successive R-peaks), called heart rate variability (HRV), or interbreath intervals (that is interval between successive breaths (IBIs)), called respiratory rate variability (RRV). Numerous methods have been developed to characterize variability mathematically. These methods have been applied in diverse clinical studies, demonstrating that healthy biological systems possess innate and highly complex patterns of variability, and illness is associated with altered variability and reduced complexity [17-20]. A decrease in variability is indicative of reduced adaptability, reflects a ‘stressed’ system [21,22], and has been described as a marker of outcome in multiple pathological states, for example sepsis [22]. We and others have hypothesized that cardiorespiratory variability might be used as a marker of the ability of the cardiopulmonary system to tolerate the increased workload associated with both an SBT, and subsequently, extubation. In several single-center studies, both HRV [23] and RRV [24,25] during SBTs have been shown to be associated with failed SBTs or extubation failure; however, the added predictive value of variability measures over and above existing methods has not yet been evaluated.

The two goals of our study were: (1) to compare variability in patients who passed and failed extubation using a wide array of HRV and RRV measures, and (2) to investigate the added value of HRV and RRV in the prediction of extubation outcomes, both individually and in combination, as compared to commonly used clinical variables, namely heart rate (HR), respiratory rate (RR), tidal volume (TV), and RSBI.

## Methods

The weaning and variability evaluation (WAVE) research study was a prospective, blinded observational multicenter cohort study conducted in 12 centers. Research ethics boards at each site waived consent for enrolment in this strictly observational study (Ottawa - Ottawa Health Science Network Research Ethics Board (OHSN-REB)). The study was powered based on preliminary data from a single-center pilot (n = 60), to estimate the fold increase of extubation failure (respect to average failure rate - that is 12% in this population) within a margin of error of 10% or less with two-sided α = 0.05.

Patients were considered for enrolment when an SBT was planned in anticipation of extubation. Inclusion criteria were: invasive mechanical ventilation for >48 hours, at least partial reversal of the condition precipitating mechanical ventilation, stabilization of other organ systems, toleration of pressure support ventilation ≤14 cm H_2_O (oxygen saturation (SpO_2_) ≥90% with fraction of inspired oxygen (FiO_2_) ≤40% and positive end-expiratory pressure (PEEP) ≤10 cm H_2_O), hemodynamic stability (low - phenylephrine <50 ug/min; norepinephrine <5 ug/min; dobutamine <5 ug/kg/min; milrinone <0.4 ug/kg/min - or no vasopressors), stable neurological status (no deterioration in Glasgow coma score during prior 24 hours and, if measured, intracranial pressure (ICP) <20 mmHg), and intact airway reflexes (cough and gag). Exclusion criteria were: order not to reintubate should the patient fail extubation, anticipated withdrawal of life support, known or suspected severe weakness (myopathy, neuropathy or quadriplegia), tracheostomy, atrial fibrillation, and prior extubation during ICU stay.

### Case report forms (CRF)

Research teams at each site screened daily to identify study participants, and completed clinical case report forms (CRFs). Respiratory therapists (RTs) performed the SBTs and completed the SBT and Extubation CRFs. The SBT CRF (one per SBT) included ventilator settings (pressure support, PEEP prior to and during the SBT, FiO_2_, average TV, and minute ventilation), RR, HR, blood pressure, SpO_2_ and RSBI at the 2 minutes, 15 minutes, 30 minutes, and end time of the SBT. The Clinical CRF (one per patient) included patient demographics, ICU admission diagnosis, comorbidities, acute physiology and chronic health evaluation II (APACHE II) on the day of admission, date and time of extubation, survival status 30 days after ICU admission, need for tracheostomy, and the need and etiology for reintubation (along with reintubation date and time). Immediately prior to extubation and following a decision to extubate, research personnel completed an Extubation CRF. This form recorded the treating team’s clinical perception of risk of extubation failure (high >15%, average 5 to 15%, low <5%), as well as factors assessed in considering the patient’s readiness for extubation. Failed extubation was defined as reintubation within 48 hours of extubation.

### Signal acquisition and processing

RTs attached CO_2_ modules to the bedside monitor and affixed CO_2_ tubing to the ventilator circuit at least 30 minutes prior to SBT. Waveform data collection included electrocardiogram (ECG) lead II and CO2 data from 30 minutes prior to the SBT until 30 minutes following its conclusion (encompassing the entire SBT).

R-peak to R-peak interval (RRI) time series were extracted from the ECG waveform using a well-known R-peak detection algorithm [26]. Ectopic beats were excluded using beat annotations as well as a threshold-based detection algorithm [27]. Similarly, the time interval between two successive breaths, that is IBI time series, was extracted from CO_2_ waveforms (125 Hz) through standard zero-crossing detection.

### Waveform quality and variability analyses

**Figure 1:**
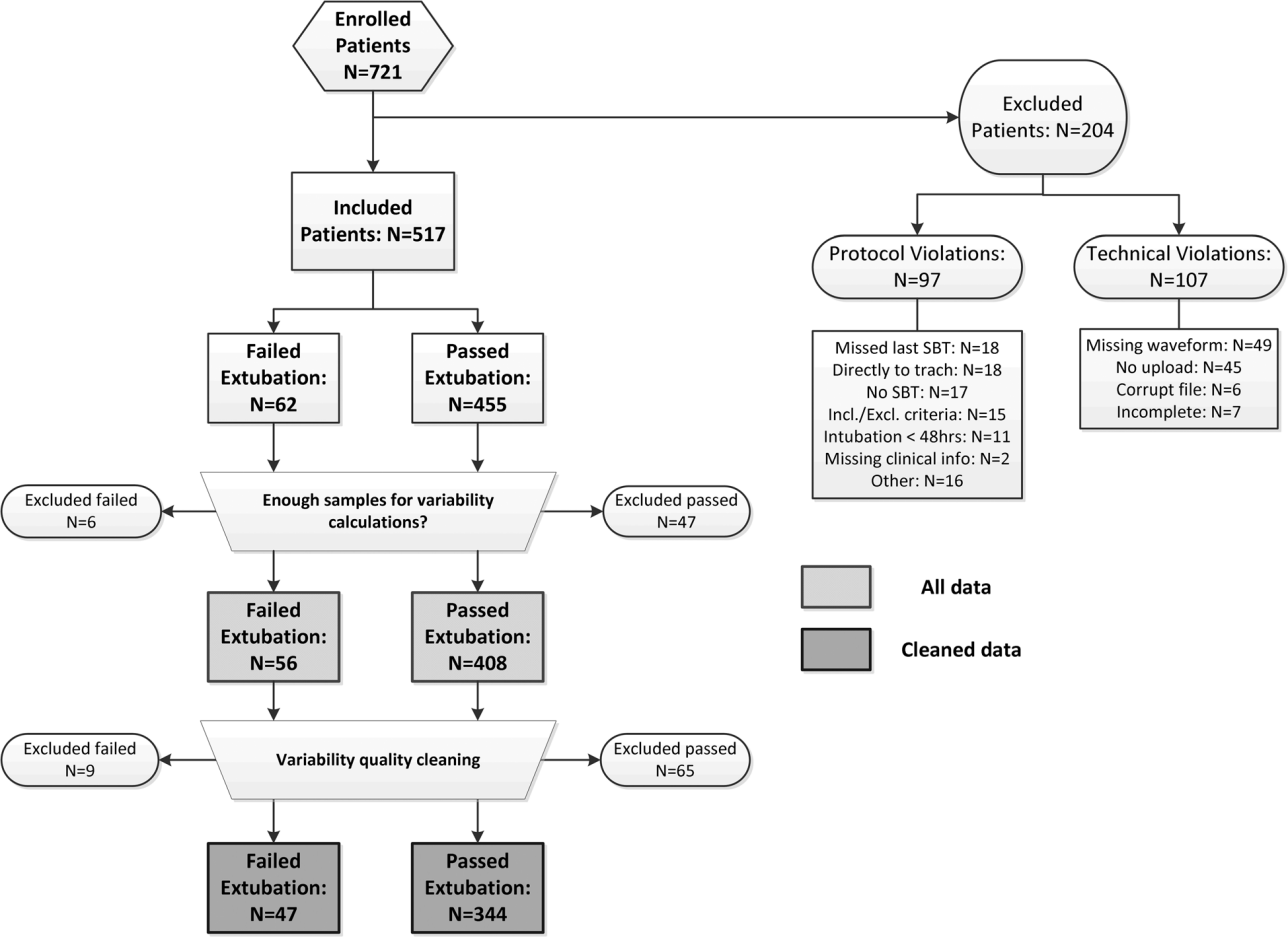
**Flow diagram of selection of patients.** Beside standard exclusions due to protocol and technical violations, the diagram shows how the dataset was reduced to ensure proper variability computation. In particular, patients were excluded when (1) having less than two windows of both heart rate and respiratory rate variability to analyze prior and during the spontaneous breathing trial, and (2) variability was extracted from waveforms deemed to be poor quality.

Using Continuous Individualized Multiorgan Variability Analysis (CIMVA™) software, a set of 97 measures of HRV and 82 measures of RRV (listed in the electronic supplement [28]) was calculated and tracked over time through a windowed analysis of data collected before and during the SBT prior to extubation (that is the last SBT). This analysis consisted of (1) taking a window of the RRI/IBI data (5 minutes for HRV and 15 minutes for RRV), (2) computing all variability measures for the given window, and (3) repeating the computation on successive windows with a step size of 2.5 minutes for both HRV and RRV. Waveform quality was assessed in an automated fashion for each window; briefly, the quality filtering was based on the morphology of the ECG/CO_2_ waveforms, the level of noise/artefacts and the proportion of disconnected/saturated periods [29,30]; this information was used to exclude patients without high-quality ECG/CO_2_ and RRI/IBI data (see Figure 1 for exclusions due to poor data quality). The outcome of the variability analysis was summarized over two intervals (30 minutes immediately prior to SBT start and the first 30 minutes of the SBT), by computing the median of each variability time series within these intervals (excluding windows that contained the SBT start time). The change in variability (defined as the median variability during the SBT minus the median variability prior to the SBT (that is delta = during to pre), was also computed.

### Statistical analysis

In addition to the HRV and RRV measures, study subjects were compared for gender, age, APACHE II score, ICU admission diagnosis, comorbidities and clinician-perceived risk of extubation failure. The chi-square test was used to compare patient proportions, and the Wilcoxon rank-sum test to compare medians, which were reported with 95% confidence intervals (CIs). The robust false discovery rate [31] was used for multiple comparison correction (fixing the rate of false positives to 5%).

### Predictive modelling

Because of its simplicity and robustness [32], we utilized an ensemble averaging of univariate logistic regressions. A univariate logistic regression is a model that takes as input a single measure of variability, and provides as output the risk of failing extubation as a number between 0 and 1. The ensemble averaging consists of taking multiple univariate logistic regressions and averaging their outputs, so as to get a more robust estimate of the risk. The output of our predictive model is called the WAVE score and it represents an estimate of the probability of extubation failure, with values closer to zero indicating lower probability and values closer to one indicating higher probability. The selection of the variables to be included in the model and the unbiased estimation of its performance required the division of the dataset in three sets - training (creation of decision thresholds), validation (for identification of the best performing set of variables) and test (for unbiased performance estimation), with the use of two validation loops (repeated random subsampling), to ensure robustness of the results [33]. The identification of the best performing variables (that is feature selection) was based on a greedy optimization on the validation set. In particular, we kept those variables maximizing the sum of two specific measures: the area under the receiver operating characteristic (ROC AUC - used to select measures with high sensitivity and specificity), and the positive predictive value (PPV - used to maximize predictive accuracy of failed extubation). The greedy optimization started from the single univariate logistic regression showing the highest (ROC AUC + PPV) on the validation set. Then, we added to the model, one by one, the univariate logistic regression improving the performance on the validation set, until five variables were selected. We imposed to use five variables following the rule of thumb that log(n) variables should be used with a dataset of n samples. Ideally, we should have run a cross-validation loop to optimize the number of variables, however, that was not suitable because of the low number of patients who failed extubation. The model was then evaluated on the test data for unbiased estimation of its performance. The described process was repeated 500 times to yield a robust estimate of the average performance of the predictive model. Please see Additional file 1 for additional information.

Subsequently, we trained the ensemble average of univariate logistic regressions on all the data and evaluated how it performed on subgroups of patients. Although the results of the model on the same data used to create it (that is training-set results) are biased, they enable the comparison of the performance across subgroups. In particular, we characterized the risk/fold increase in risk of failing extubation in four subgroups: low vs. high RSBI (threshold of 105 breaths/min/L, consistent with prior studies [11,34]), and low or average vs. high clinician-perceived risk of failure.

## Results

We enrolled 721 patients, 60 patients between November 2007 and April 2009 in a run-in pilot, and the remaining 661 between November 2009 and December 2012. See Figure 1 for a flow diagram of the patient selection process. After exclusions, 434 subjects remained (51 (approximately 12%) failed and 383 passed). These 434 subjects constituted the cohort undergoing CIMVA analysis. The ‘failed’ and ‘passed’ included and excluded populations were similar, other than the proportion of patients assessed as having low/average/high risk of failing extubation and the values of RSBI and RR at 30 minutes during the SBT (Table 1).

**Table 1:** Patient demographics

	**Passed extubation**	**Failed extubation**	***P*** value*
	**(N = 383)**^**^**^	**(N = 51)**^**^**^	
Gender:			
Males, n (%)	186 (48.6)	29 (56.7)	0.27
Females, n (%)	191 (49.9)	21 (41.2)	0.24
Age (95% CI)	63 (61, 64)	65 (58, 69)	0.86
APACHE II score (95% CI)	19 (19, 20)	20 (18, 23)	0.21
Level of sedation^x^ (95% CI)	0 (0, 0)	0 (−1, 0)	0.78
ICU admission diagnoses			
Cardiovascular, n (%)	112 (25.4)	12 (20.0)	0.40
Respiratory, n (%)	87 (19.7)	18 (30.0)	0.05
Infections, n (%)	62 (14.1)	10 (16.7)	0.54
Gastrointestinal, n (%)	34 (7.7)	3 (5.0)	-
Surgery, n (%)	33 (7.5)	3 (5.0)	-
Head, n (%)	36 (8.2)	1 (1.7)	-
Renal, n (%)	18 (4.1)	2 (3.3)	-
Trauma, n (%)	8 (1.8)	1 (1.7)	-
Overdose, n (%)	9 (2.0)	1 (1.7)	-
Pancreatitis, n (%)	3 (0.7)	1 (1.7)	-
Hepatobiliar, n (%)	5 (1.1)	0 (0.0)	-
Other, n (%)	34 (7.7)	8 (13.3)	0.12
Comorbidities^+^:			
None, n (%)	237 (61.9)	28 (54.9)	0.34
Lung, n (%)	90 (23.5)	15 (29.4)	0.35
Heart, n (%)	81 (21.1)	13 (25.5)	0.48
Both, n (%)	25 (6.5)	5 (9.8)	0.39
Ventilation settings pre-SBT:			
PEEP (95% CI) (cm H2O)	10 (8 10)	8 (8 10)	0.90
PS (95% CI) (cm H2O)	10 (10 10)	10 (10 10)	0.14
FiO_2_, (95% CI)	30 (30 30)	30 (30 30)	0.04
Ventilation settings during SBT:			
PEEP (95% CI) (cm H2O)	5 (5 5)	5 (5 5)	0.46
PS (95% CI) (cm H2O)	5 (5 5)	5 (5 5)	0.01
FiO_2_, (95% CI)	30 (30 30)	30 (30 30)	0.11
Perceived risk of failure:			
N/A, n (%)	53 (13.8)	7 (13.7)	0.98
Low, n (%)	117 (30.5)	6 (11.7)	0.005
Average, n (%)	180 (47.0)	26 (51.1)	0.59
High, n (%)	33 (8.7)	12 (23.5)	0.001
Respiratory rate: [breaths/min]			
Pre-SBT (95% CI)	16.0 (16.0, 18.0)	18.0 (15.0, 22.0)	0.09
During SBT (95% CI)	18.4 (17.9, 19.0)	21.7 (18.7, 25.0)	0.005
RSBI: [breaths/min/L]			
Pre-SBT (95% CI)	34.1 (31.8, 36.4)	40.0 (32.5, 50.0)	0.16
During SBT (95% CI)	42.7 (39.3, 45.6)	46.6 (40.0, 67.5)	0.005

Low, average and high-risk categories were based on clinical impression. ^^^There is a maximum amount of 2% of missing values in each category, due to clinical data not recorded (for example males and females in the failed population add up to 50, instead of 51); *comparison between passed and failed using Wilcoxon rank-sum test to compare the medians, or chi-square test to compare the proportions (no *P* value was provided for those variables with less than five samples); ^x^computed through Richmond agitation-sedation scale (RASS) score; ^+^heart comorbidities are coronary artery bypass graft, dilated cardiomyopathy, congestive heart failure, and coronary artery disease; lung comorbidities are pulmonary fibrosis, chronic obstructive pulmonary disease (COPD), asthma; ‘None’ corresponds to no lung or heart comorbidities. APACHE II, acute physiology and chronic health evaluation II; ICU, intensive care unit; SBT, spontaneous breathing trial; PEEP, positive end-expiratory pressure; PS, pressure support; FiO_2,_ fraction of inspired oxygen.

**Figure 2:**
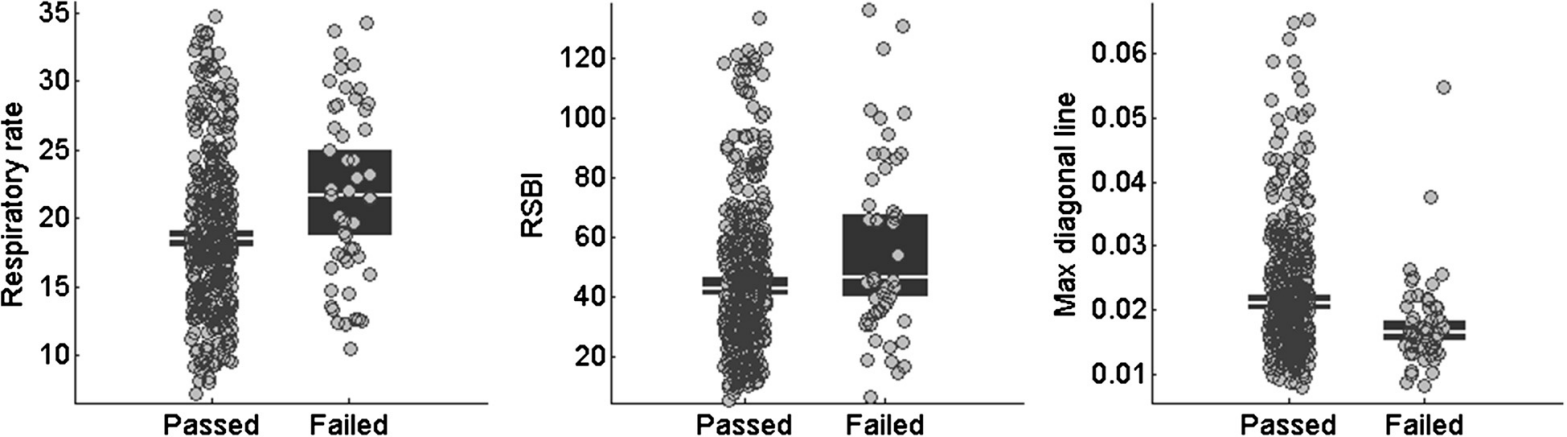
**Distributions of respiratory rate (RR),rapid shallow breathing index (RSBI) and variability.** This figure shows the distribution of values for passed and failed of three different measures (from the left: respiratory rate, rapid shallow breathing index, and respiratory rate variability recurrence quantification analysis: maximal diagonal line). Each grey circle represents a subject. The black box with a white line in between represents the median with its 95% confidence interval.

None of the variability measures computed prior to the SBT, nor the difference between variability measures during and prior the SBT, were found to be statistically significant, when adjusted for multiple comparisons using a false discovery rate of 5%. Only the variability measures calculated during the SBT were found significantly associated with extubation failure. As a result, only during SBT variability measures were considered in the subsequent predictive model analysis. Ten measures of variability (two HRV and eight RRV) during the SBT were statistically significant (*P* <0.0041 - threshold with 5% false positives), as summarized in Table 2. A visual representation of the distributions for RR, RSBI and the measure of variability with the lowest *P* value (that is RRV recurrence quantification analysis: maximal diagonal line) is provided in Figure 2. Despite the low *P* values associated with univariate comparisons, the distributions manifest substantial overlap between the passed and failed categories, highlighting the need for a multivariate predictive model.

**Table 2:** Statistically significant comparisons of during spontaneous breathing trial (SBT) variability

**Variability domain**	**Measure name**	**Passed (n = 383)**	**Failed (n = 51)**	***P*** **value**
Statistical	HRV Mean of the differences	1.4 10^−6^ (−9.4 10^−7^, 3.7 10^−6^)	−8.4 10^−6^ (−1.5 10^−5^, −1.9 10^−6^)	0.00278
Geometric	RRV Recurrence quantification analysis: average diagonal line	0.0057 (0.0054, 0.0060)	0.0044 (0.0038, 0.0053)	0.00011
	RRV Recurrence quantification analysis: maximum diagonal line	0.021 (0.020, 0.022)	0.016 (0.015, 0.018)	0.00004
	RRV Recurrence quantification analysis: maximum vertical line	0.017 (0.016, 0.018)	0.012 (0.011, 0.014)	0.00017
	RRV Recurrence quantification analysis: trapping time	0.0048 (0.0046, 0.0050)	0.0038 (0.0030, 0.0042)	0.00009
Informational	RRV Fano factor distance from a Poisson distribution	−0.12 (−0.12, −0.11)	−0.15 (−0.17, −0.12)	0.00166
Energetic	RRV Hjorth parameters: activity	11.1 (10.4, 11.8)	7.8 (6.0, 10.7)	0.00406
Invariant	HRV Power Law (based on frequency) x intercept	15.8 (14.8, 17.3)	10.0 (4.5, 13.9)	0.00255
	RRV Largest Lyapunov exponent	1.02 (1.00, 1.02)	1.07 (1.03, 1.14)	0.00151
	RRV Power Law (based on histogram) y intercept	−2.17 (−2.21, −2.10)	−2.35 (−2.59, −2.15)	0.00259

For specific details about each measure, refer to [28]. HRV, heart rate variability; RRV, respiratory rate variability.

The comparison of WAVE score (unbiased test set performance results) with logistic regression models based on clinical parameters commonly used to predict extubation outcome is reported in Table 3, showing that RRV variables achieved the highest ROC AUC, demonstrating improved sensitivity (+25%), without substantial changes in PPV (identical) or negative predictive value (NPV) (+3%).

**Table 3:** Prognostic accuracy comparison

**Model**	**ROC AUC**	Sensitivity*	Specificity*	PPV*	NPV*	NRI*
Single logistic regression: heart rate	0.51	0.5	0.55	0.11	0.87	0.22
Single logistic regression: RSBI	0.61	0.5	0.72	0.18	0.91	0.04
Single logistic regression: respiratory rate	0.63	0.5	0.66	0.17	0.91	0.04
Ensemble of three univariate logistic regressions:	0.62	0.5	0.69	0.17	0.90	0.07
Heart rate, respiratory rate, RSBI						
WAVE score	0.69	0.75	0.59	0.18	0.94	-

*Positive test for probability of failure equal or above 0.5. ROC AUC, area under the receiver operating characteristic curve; PPV, positive predictive value; NPV, negative predictive value; NRI, net reclassification improvement; RSBI, rapid shallow breathing index; WAVE, weaning and variability evaluation.

**Figure 3:**
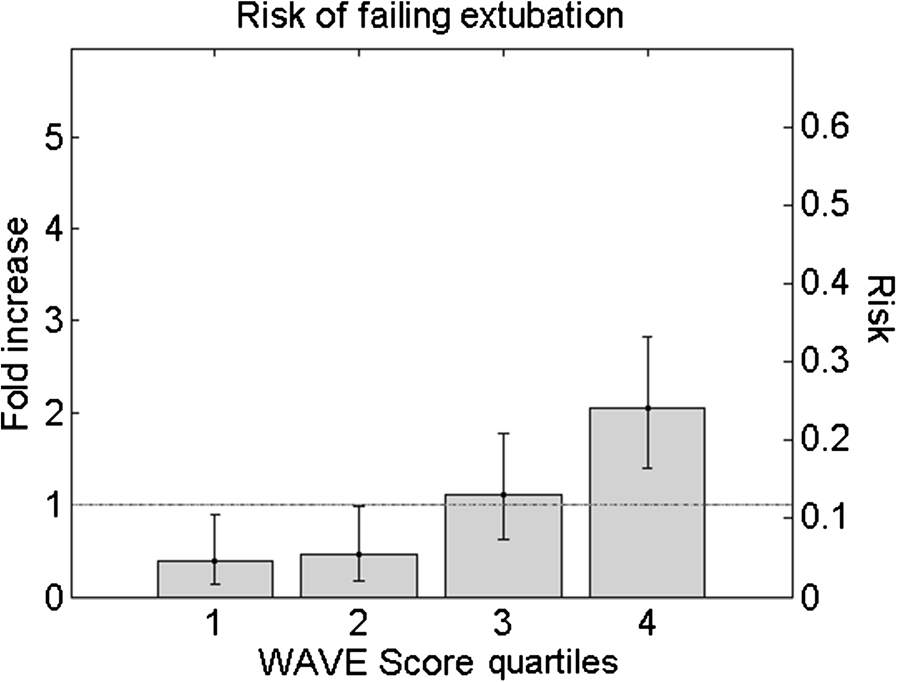
**Weaning and variability evaluation (WAVE) score quartile.** This figure shows the risk/fold increase in risk of failing extubation associated with each quartile of the population. The risk is defined as the number of patients who failed divided by the total number of patients in a given quartile. The fold increase in risk is the risk divided by the average risk of failure of the dataset (approximately 12%). The total number of patients is 434, therefore each quartile is representative of 108 patients.

**Figure 4:**
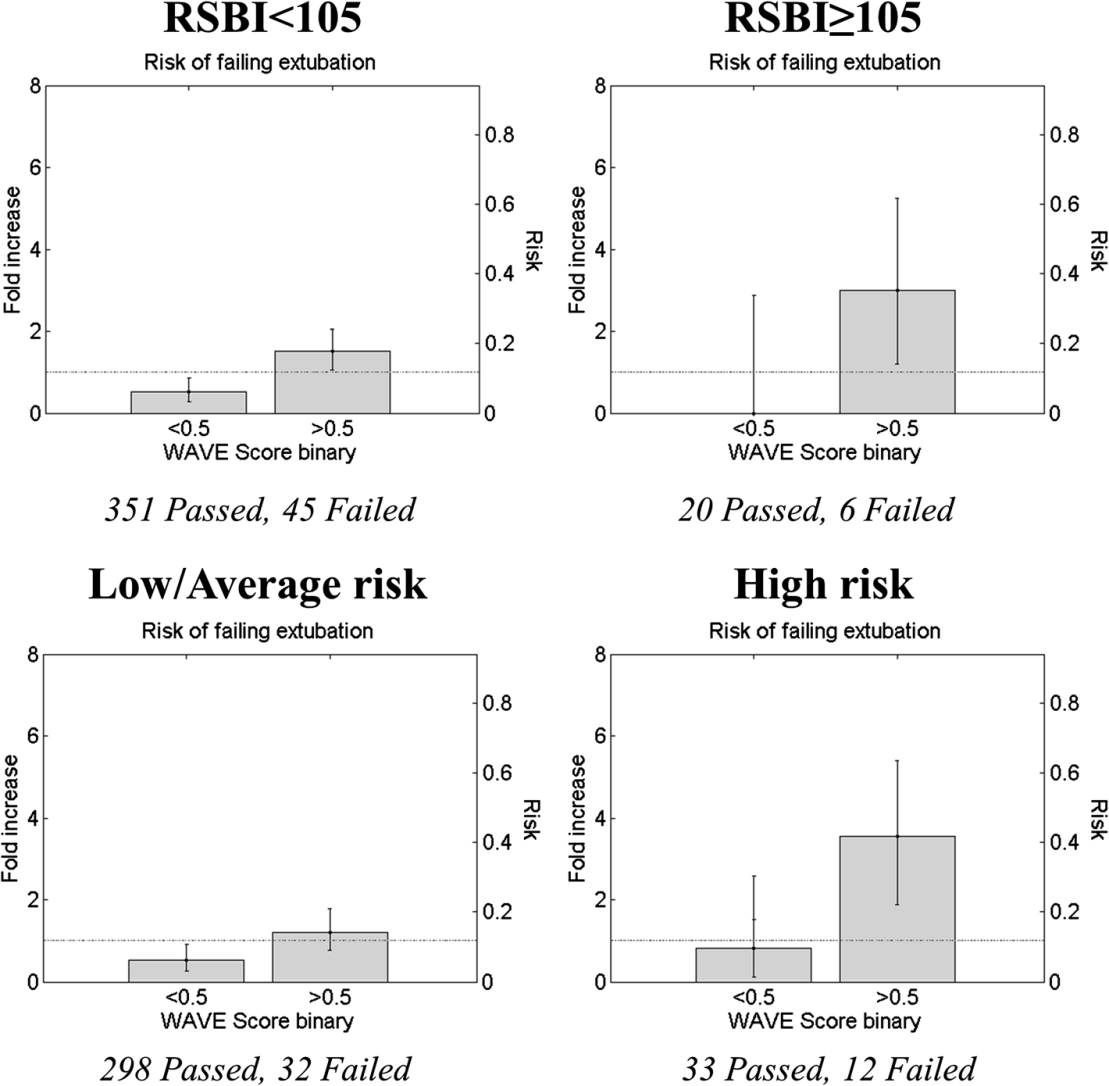
**Weaning and variability evaluation (WAVE) score, rapid shallow breathing index (RBSI) and clinical impression.** These figures show how the risk/fold increase in risk of failing extubation associated with positive WAVE score (that is above 0.5) increases with increasing RSBI during SBT (above), or the clinical impression of the physician at the end of the SBT (below). The risk is defined as the number of patients who failed divided by the total number of patients in a given group (for example, above 0.5). The fold increase in risk is the risk divided by the average risk of failure of the dataset (approximately 12%). There are 396 patients with low RSBI (45 failed, 351 passed), and 26 patients with high RSBI (6 failed, 20 passed), while 12 passed had no RSBI reported. There is no statistically significant difference between the number of failed and passed that had no RSBI reported (*P* value = 0.2, chi-squared test for proportions). There are 330 patients with low/average risk of failure (32 failed, 298 passed), and 45 with high risk of failure (12 failed, 33 passed), while 7 failed and 52 passed have no perceived risk of failure reported. There is no statistically significant difference between the number of failed and passed that had no perceived risk of failure reported (*P* value = 0.98, chi-squared test for proportions).

We further characterized the WAVE score by assessing its training-set performances (1) in the whole population stratified in quartiles, and (2) in association with RSBI and clinical impression of perceived extubation risk, using a decision threshold of 0.5 on the WAVE score. The risk of failure was defined as the number of patients who failed divided by the total number of patients in a given group. The fold increase in risk is the risk divided by the average risk of failure of the dataset (approximately 12%). The training set performances on the entire dataset (that is no data left in the test set) stratified in quartiles are shown in Figure 3, where we see that for higher WAVE scores there is a corresponding higher risk of failing extubation. Similarly using a binary cutoff, we found a fold increase in risk for WAVE score above 0.5 of 1.59 (95% CI: 1.16, 2.02). In Figure 4, we observed that the higher the RSBI, or the clinician-perceived risk of failure, the stronger the ability of the model to identify extubation failure. In particular, the fold increase in risk for WAVE score above 0.5 moved from 1.5 (CI: 1.05, 2.04) for patients with RSBI <105 to 3.00 (CI: 1.21, 5.24) for patients with RSBI >105. Similarly, the risk increased from 1.21 (CI: 0.77, 1.78) for patients with a low/average perceived risk of failure to 3.54 (CI: 1.88, 5.38) for those having a high perceived risk of failure. We performed the same comparison on the ROC AUCs. The ROC AUC of the WAVE score on the entire dataset was 0.72. For the subgroups we achieved instead (in order): 0.67 for patients with low/average perceived risk of failure, 0.69 for those with low RSBI, 0.82 for patients with high perceived risk of failure, 0.87 for those with high RSBI (*P* <0.01 and *P* = 0.09, respectively).

## Discussion

This multicenter observational study demonstrates that in a broadly heterogeneous population of critically ill patients requiring mechanical ventilation, both HRV and RRV during the last SBT prior to extubation are associated with subsequent extubation failure. Two measures of HRV and eight measures of RRV recorded during the last SBT prior to extubation showed statistically significant differences with respect to extubation outcome, and to a greater degree than HR, RR or RSBI.

Using a machine learning analysis with randomized repeated subsampling and cross-validation, the average predictive capacity of RRV during the last SBT was superior to all other measures. The use of several measures of RRV to derive the WAVE score showed a higher ROC AUC, outperforming RR, HR and RSBI. Standard choices for the parameters of the repeated random subsampling validation (80% training, 10% validation, 10% test) [33] and the decision threshold to compute sensitivity, specificity, negative/positive predictive value (threshold = 0.5) were chosen to reduce bias. The training-set performances of the WAVE score were used to evaluate subgroup performances to get a sense of the way the model might be used clinically. The ROC AUC was 0.87 and 0.82 in the high-risk patients, based on RSBI >105 and high perceived risk of extubation failure, respectively. In contrast to the average unbiased model performance, these performances are biased because they are tested on the entire dataset from which the WAVE score was derived (that is trained and validated). Taken together, the results demonstrate that RRV during the last SBT outperforms any other measure(s) to predict extubation outcomes, and the subgroup analyses highlight the potential utility and complementarity of the WAVE model for extubation decision making.

The study was inclusive of a highly heterogeneous group of patients (including a wide variation in age, ICU admission diagnosis and comorbidities) in an observational study with absence of strict protocolization of SBT performance (for example type, pressure support levels, duration) or extubation decision making; thus, the variation in patients and practice may have diluted the observed signal.

As extubation is associated with an increase in work of breathing [35], and extubation failure is commonly due to the inability of the cardiorespiratory system to tolerate this increased workload, it is not surprising that the commonly utilized quantitative tests for predicting failed extubation [12] (for example RSBI) are markers of inability of the cardiorespiratory system to respond to an increased workload. We hypothesized that variability monitoring would improve the ability to detect stress and inability to tolerate the increased workload of breathing associated with an SBT, and subsequently, extubation. Our findings support this hypothesis, and are consistent with prior studies that have demonstrated that HRV and RRV help to predict SBT or extubation outcomes. In 2001, El-Khatib *et al*. showed that during the SBT, 13 patients who failed extubation (defined as reintubation within 24 hours) had lower complexity than 39 patients who were successfully extubated [36]. In 2003 Shen *et al*. [23] showed that spectral measures of HRV were reduced in 12 patients who failed the weaning trial (either failing extubation or not being ready for extubation). Bien *et al*. [24] showed in 78 postoperative patients (57 passed weaning, 21 failed) that four measures of RRV, along with RR and RSBI, exhibited significant differences between the two groups. Work by Wysocki *et al*. in 2006 [25] confirmed the results of Bien in 46 ICU patients (32 passed and 14 failed). Papaioannou *et al*. [37] published a study on 42 postoperative patients (24 passed weaning, 18 failed), showing that a set of nonlinear measures of HRV and RRV provided added value to a predictive model based on RSBI.

The understanding of altered respiratory rate dynamics in association with extubation failure remains an area of active investigation. Of note, the recurrence quantification analysis (RQA) of RRV emerged as highly significantly associated with extubation failure (Table 1). The recurrence plot is a technique that projects a time series (in this case the IBI time series) in a higher dimensional space, called phase space. In that space, the pairwise Euclidean distance between all points is computed, creating a matrix where each row and each column is a point in the phase space, and each element of the matrix is the respective distance. When this distance is smaller than a given threshold, that is two points are close in the phase space, a ‘recurrence’ occurs. RQA consists in the study of the number and types of recurrences that appear in a recurrence plot. Failed extubation appeared to show slightly higher degree of chaotic dynamics, given by the shorter length of diagonal and vertical lines, as compared to passed extubation. This result is supported by an increase in the largest Lyapunov exponent, a measure of chaoticity of a system. These findings are in keeping with shorter RQA diagonal lines demonstrated during 39 failed SBTs compared to 92 successful SBTs (albeit with no study of extubation) [38]. For more details on these measures, refer to [17].

Given that prior studies utilized visual inspection of waveform data to ensure adequate waveform quality, an important strength of this study was the use of automated quality filters. No visual inspection or hand-selected methodology was utilized to choose waveforms or patients for variability analyses. It is well known that artifact, ectopy and nonstationarity can dramatically alter variability measures [39]. Our quality filters were developed based on published ECG-quality algorithms [29,40], as well as proprietary capnography-quality filters trained with separate datasets. We verified *a posteriori* that the predictive performance of the model described in this study was significantly lower when including poor quality waveform data.

There are several important limitations to this study, the most significant being its observational derivational design. As such, there was no strict protocolization regarding the way SBTs were performed, nor regarding decisions about extubation. This was a pragmatic observation of a heterogeneous group of patients being considered for extubation. The majority of patients (75.4%) had a ventilator setting of 5 cm H_2_O pressure support and 5 cm H_2_O PEEP during the SBT, which may diminish the variability signal (compared to T-piece SBT), reducing specificity [41] and dampening the observed signal within this study. By limiting our analysis to the last SBT preceding extubation, we have utilized the information available to clinicians making the decision to extubate; however there may be information utilized by physicians in following SBT results from day to day, which were not captured in this study. Just over half of all patients were enrolled in a single center, limiting external generalizability; although no significant differences were observed in SBT ventilator settings or model performance of the single site (ROC AUC 0.72) compared to all others (ROC AUC 0.74), a more even distribution of enrolment in a validation cohort is required. A large number of patients were excluded from the analyses, highlighting the challenge in obtaining waveform data, and the potential for a patient to deviate from expected planned extubation. The exclusions appeared random, occurred throughout the study, and equally from all sites; we did not detect any systematic pattern to the exclusions, either technical or protocol violations, or poor waveform quality. Lastly, although to create the WAVE score we made multiple choices to maximize its generalizability - such as using an ensemble of logistic regressions rather a single multivariate, using stratified cross-validation, choosing a nonoptimized number of features (that is five measures of variability), and using the generic decision threshold of 0.5 - there is no guarantee that we did not overfit the predictive model to our population, particularly because of the small number of patients who failed extubation. External validation of the WAVE score is required.

The question of the incremental value of the WAVE score is critically important and complex. Clinical experience and the data shown in this manuscript highlight that extubation outcome prediction is difficult, and no test in isolation is capable of determining extubation outcomes. It is a fully integrated assessment made by ICU clinicians that determines the optimal timing of extubation, based on the assessment of SBT performance, clinical trajectory, comorbidities that affect the risk of harm of extubation failure, patient/family wishes/goals of care and other potential factors. No clinician evaluates a single score to make this complex decision, and the WAVE score is not intended to be used in isolation as a determinant of extubation outcomes. Nonetheless, we believe, that the WAVE score, when used in conjunction with existing measures (for example RSBI) provides optimal prediction of extubation outcomes that will be beneficial to the decision-making process. For example, the 75% sensitivity of the WAVE score model is higher than the 50% sensitivity of RSBI that we observed in our study. The augmented ROC AUC in high-risk patients suggest the complementary utility of avoiding unnecessary delays in extubation when the WAVE score is less than 0.5 or considering alternatives to extubation if the WAVE score is greater than 0.5 in patients deemed high risk based on traditional measures. The patients conventionally identified as high risk may well be the ones who benefit most from the additional WAVE test. In general, we showed that the WAVE score can stratify patients in multiple categories of risk (Figure 4), thereby providing clinicians with a more representative picture of the status of a patient. The ultimate aim of this research program is to introduce extubation clinical decision support immediately following SBT completion. We anticipate that combining (1) a standardized method of performing and assessing an SBT, (2) conventional predictive measures, and (3) a novel score like WAVE, will optimally assist the clinician in the decision to extubate. Following a validation study, the true incremental value of this approach would be assessable in a randomized controlled trial.

## Conclusions

The determination of optimal timing for extubation of critically ill patients remains an integrated clinical evaluation and assessment made by a clinician at the bedside, with the full understanding of that patient’s goals of care, past medical history, etiology of respiratory failure, clinical course in the ICU, as well as their performance on their last SBT prior to extubation. In the largest multicenter study to evaluate the potential and added value of variability in assisting with assessing extubation readiness, we have found that altered HRV and RRV during the last SBT prior to extubation are significantly associated with extubation failure, and a predictive model derived from RRV during SBT provides added prognostic accuracy in predicting extubation failure when compared to physiological variables used in clinical practice, particularly in high-risk patients. This model requires validation in an independent cohort to verify its generalizability, and a randomized trial to assess its clinical utility.

## Key messages

● No single measure drawn from SBT performance is capable of accurately predicting extubation outcomes; extubation outcome prediction is challenging.

● Altered HRV and RRV during the last SBT prior to extubation are associated with subsequent extubation failure.

● A multivariate predictive model based on RRV during the last SBT offers improved predictive accuracy of extubation outcomes compared to physiological variables commonly used in clinical practice, particularly in patients deemed high risk of failure based on traditional measures.

● A multicenter validation study is merited and necessary to evaluate the accuracy of the derived predictive model.

### Consent

Written informed consent was obtained from the patients for the publication of this report and any accompanying images.

## Abbreviations

APACHE II: acute physiology and chronic health evaluation II; AUC: area under the curve; CI: confidence interval; CIMVA: continuous individualized multiorgan variability analysis; COPD: chronic obstructive pulmonary disease; CRF: case report form; ECG: electrocardiogram; FiO2: fraction of inspired oxygen; HR: heart rate; HRV: heart rate variability; IBI: interbreath interval; ICU: intensive care unit; MEP: maximal expiratory pressure; MIP: maximal inspiratory pressure; NPV: negative predictive value; PEEP: positive end-expiratory pressure; PPV: positive predictive value; ROC: receiver operating characteristic; RQA: recurrence quantification analysis; RR: respiratory rate; RRI: R-peak to R-peak interval; RRV: respiratory rate variability; RSBI: rapid shallow breathing index; RT: respiratory therapist; SBT: spontaneous breathing trial; SpO2: oxygen saturation; TV: tidal volume; WAVE: weaning and variability evaluation.

## Competing interests

Andrew Seely is Founder and Chief Science Officer of Therapeutic Monitoring Systems (TMS); TMS aims to commercialize patent-protected applications of multiorgan variability monitoring to provide variability-directed clinical decision support at the bedside to improve care for patients at risk for or with existing critical illness. Andrew Seely holds a patent jointly with co-authors Andrea Bravi and André Longtin on composite measures of variability. Geoffrey Green is Product Manager for TMS. John Marshall and Gari Clifford are on the Scientific Advisor Board of TMS. Other authors have no relevant conflict of interest to disclose.

## Authors’ contributions

AS conceived of the study, and led the grant submissions, study design, recruitment, analyses and writing. AB led the statistical and variability analyses, assisted by CH, GG, AL, TR, GR and DF. DK participated in drafting the protocol, enrolling patients, and participated in the analysis. DM, TR, DF, LM, GR, GC and NF all provided input into the design, analyses, along with manuscript review and editing. JM, SM, FJ, SB and CM all participated in patient enrolment, as well as providing input regarding design and analyses. AF was the clinical research coordinator for the study, interfacing with all sites, coordinating REB submissions. AF also contributed to the protocol, and directly coordinated the multicenter conduct of the study. All the authors were involved with the revision of the manuscript and provided final approval.

## Supplementary Material

Additional file 1: This file describes in great detail how the predictive model was developed and how unbiased performance was estimated. Click here for file
