# Erratum to: Do heart and respiratory rate variability improve prediction of extubation outcomes in critically ill patients?

**DOI:** 10.1186/s13054-014-0620-z

**Published:** 2014-12-02

**Authors:** Andrew JE Seely, Andrea Bravi, Christophe Herry, Geoffrey Green, André Longtin, Tim Ramsay, Dean Fergusson, Lauralyn McIntyre, Dalibor Kubelik, Donna E Maziak, Niall Ferguson, Samuel M Brown, Sangeeta Mehta, Claudio Martin, Gordon Rubenfeld, Frank J Jacono, Gari Clifford, Anna Fazekas, John Marshall, The Canadian Critical Care Trials Group (CCCTG)

**Affiliations:** Ottawa Hospital Research Institute, 725 Parkdale Avenue, Ottawa, ON K1Y 4E9 Canada; University of Ottawa, 75 Laurier Avenue East, Ottawa, ON K1N 6N5 Canada; University Hospital Network, University of Toronto, 190 Elizabeth Street, Toronto, ON M5G 2C4 Canada; Intermountain Medical Center (IMC), Shock Trauma ICU, 5121 Cottonwood Street, Murray, UT 84157 USA; Mt Sinai, University of Toronto, 600 University Avenue, Toronto, ON M5G 1X5 Canada; London Health Sciences Center, 339 Windermere Road, London, ON N6G 2V4 Canada; Sunnybrook Hospital, University of Toronto, 2075 Bayview Avenue, Toronto, ON M4N 3M5 Canada; University Hospital Case Medical Center, Case Western Reserve University, 11100 Euclid Avenue, Cleveland, OH 44106 USA; University of Oxford, Kellogg College, Banbury Road, Oxford, OX2 6PN United Kingdom; St. Michaels Hospital, University of Toronto, 30 Bond Street, Toronto, ON M5B 1W8 Canada; Divisions of Thoracic Surgery & Critical Care Medicine, 501 Smyth Road, Ottawa, ON K1H 8L6 Canada

## Erratum

While compiling this article [1] one of the authors was inadvertently omitted from the author list. This author, The Canadian Critical Care Trials Group (CCCTG), has been included in the corrected author list above.

## References

[1] Seely AJE, Bravi A, Herry C, Green G, Longtin A, Ramsay T, Fergusson D, McIntyre L, Kubelik D, Maziak DE, Ferguson N, Brown SM, Mehta S, Martin C, Rubenfeld G, Jacono FJ, Clifford G, Fazekas A, Marshall J (2014). Do heart and respiratory rate variability improve prediction of extubation outcomes in critically ill patients?. Crit Care.

