# Supplementary material for: Do heart and respiratory rate variability improve prediction of extubation outcomes in critically ill patients?
Source: Crit Care. 2014 Apr 8;18(2):R65. doi: 10.1186/cc13822 (PMC4057494; doi:10.1186/cc13822)
Supplement: Additional file 1: — This file describes in great detail how the predictive model was developed and how unbiased performance was estimated. [file cc13822-S1.docx]

**Do Heart and Respiratory Rate Variability**

**Improve Prediction of Extubation Outcomes**

**in Critically Ill Patients?**

Andrew JE Seely, Andrea Bravi, Christophe Herry, Geoffrey Green, André Longtin, Tim Ramsay, Dean Fergusson, Lauralyn McIntyre, Dalibor Kubelik, Donna E. Maziak, Niall Fergusson, Samuel M Brown, Sangeeta Mehta, Claudio Martin, Gordon Rubenfeld, Frank J Jacono, Gari Clifford, Anna Fazekas, John Marshall

**Online Data Supplement**

We developed a predictive model to forecast extubation outcomes based on five RRV variables measured during the patient’s SBT. The data set used to develop the model consisted of 82 RRV measures from 434 patients – 51 failed extubations (FE) + 383 passed extubations (PE). The following describes how the model was created and unbiased performances were estimated.

**Model development and unbiased performance estimation**

This step involved a series of 100 iterations, each of which identified a unique model consisting of five univariate logistic regressions (LR) combined in an ensemble (as described below). Each iteration involved randomly splitting the data into two sets:

1. a training and validation set (comprising 90% of the data, stratified by extubation outcome – 345 PE and 46 FE). This set was used to identify the five RRV measures to be used in the model for that iteration, as described below.
2. a test set (comprising the remaining 10%, stratified by extubation outcome – 38 PE and 5 FE). This set was used to assess the predictive performance of the model in a set of data unseen during training and validation.

For each of these 100 iterations, the following steps were performed:

1. Univariate LR model development and performance characterization

During this step, the training and validation set was further split into a separate training set (including 35 PE and 35 FE since LR requires a training set balanced by outcome) and validation set (including the remaining 310 PE and 11 FE). The training data were used to construct 82 univariate LRs (one for each RRV measure), each characterized by two parameters. These LRs were then used to derive a set of extubation failure probabilities (82, one for each RRV measure) for each patient in the validation set. This process was repeated 500 times (each resulting in a unique training/validation split, unique univariate LR parameters, and a unique set of extubation failure probabilities for the patients in the validation sets). At each iteration, the extubation failure probability for all patients in the validation set were calculated, and then used to derive area under the receiver operating curve (ROC AUC) values, positive predictive values (PPV) and sensitivities (Sens), with a probability of > 0.5 representing prediction of extubation failure.

1. Selection of variables for ensemble model using greedy optimization

The RRV measure yielding the highest median value of ROC AUC + min(PPV, Sens) (median being calculated over the 500 iterations mentioned above) was selected as the one with the best classification performance and added to the ensemble model. Following this, the other RRV measures are assessed to determine the increase in predictive power that they impart when combined in ensemble with the current model, and the RRV measure that yielded the highest performance gain is added to the model. This process (known as greedy optimization) is repeated until the model includes five RRV measures. An arithmetic average of the extubation failure probabilities is used at each stage to perform the ensemble operation.

1. Training of ensemble model

Having identified the five RRV measures, the parameters of the model are determined by training five new univariate LRs. In this case, both *training and validation sets* are used in training (unlike above, where only the training set was used). Training proceeds by including all 46 FE, and randomly selecting 46 PE (from the 345 available) to train the LRs. This is repeated 500 times (each iteration resulting in a unique set of 46 PE to be used in training and therefore, unique LR parameters). The candidate model is then formed by taking the median of the 10 LR parameters (two for each of the five RRV measures) over these 500 iterations.

1. Performance characterization on test set

Finally, the predictive performance of the ensemble model is assessed in the test set (data withheld during training and validation). This is done using a process similar to that described in i above – calculation of five extubation failure probabilities (one for each measure), ensembling of results, followed by calculation of ROC AUC and other metrics (PPV, NPV, sensitivity and sensitivity).

Following completion of this step, we are left with a distribution that illustrates the range of expected performance that can be achieved using the type of model proposed (ensemble averaging of five univariate logistic regressions). The median of this distribution is 0.69.
